# Prophylactic Olanzapine Versus Placebo in Comatose Survivors of Out‐of‐Hospital Cardiac Arrest: Protocol for a Multicenter Randomized Comparison Within the Danish Out‐of‐Hospital Cardiac Arrest (DANOHCA) Trial

**DOI:** 10.1111/aas.70289

**Published:** 2026-06-23

**Authors:** Benedikte Liebetrau, Anders M. Grejs, Martin A. S. Meyer, Lars P. K. Andersen, Sören Möller, Simon Mølstrøm, Jacob E. Møller, Henrik Schmidt, Steffen Christensen, Jesper Kjærgaard, Christian Hassager, Bodil S. Rasmussen, Jo B. Andreasen

**Affiliations:** ^1^ Department of Anesthesiology and Intensive Care Odense University Hospital Odense Denmark; ^2^ Department of Clinical Research University of Southern Denmark Odense Denmark; ^3^ Department of Cardiology Copenhagen University Hospital—Rigshospitalet Copenhagen Denmark; ^4^ Department of Intensive Care Medicine Aarhus University Hospital Aarhus Denmark; ^5^ Department of Clinical Medicine Aarhus University Aarhus Denmark; ^6^ Department of Clinical Medicine University of Copenhagen Copenhagen Denmark; ^7^ Department of Anesthesiology Zealand University Hospital Koege Denmark; ^8^ Epidemiology, Biostatistics and Biodemography, Department of Public Health University of Southern Denmark Odense Denmark; ^9^ Department of Cardiothoracic Anesthesiology Copenhagen University Hospital—Rigshospitalet Copenhagen Denmark; ^10^ Department of Anesthesiology and Intensive Care Aalborg University Hospital Aalborg Denmark; ^11^ Department of Clinical Medicine, Faculty of Medicine Aalborg University Aalborg Denmark

**Keywords:** delirium prevention, olanzapine, out‐of‐hospital cardiac arrest, postresuscitation care, randomized clinical trial

## Abstract

**Background:**

Delirium is a frequent complication in comatose Out‐of‐Hospital Cardiac Arrest (OHCA) survivors requiring postresuscitation intensive care and is associated with higher mortality and longer hospitalization. Despite this, there is no established pharmacologic strategy for delirium prevention in OHCA survivors. This trial aims to evaluate if prophylactic use of olanzapine in comatose OHCA patients can reduce delirium incidence, thereby leading to a shorter duration of hospitalization.

**Methods:**

The Danish Out‐of‐Hospital Cardiac Arrest (DANOCHA) trial is an investigator‐initiated, multicenter, randomized, controlled trial evaluating four postresuscitation interventions in adult patients resuscitated after OHCA including administration of prophylactic olanzapine compared with placebo. Comatose adult patients with OHCA of presumed cardiac cause and sustained return of spontaneous circulation (> 20 min) will be randomized 1:1 to receive either 10 mg of olanzapine or placebo immediately after inclusion in the trial and the two following evenings for a total of three doses. The primary outcome is days alive and out of the hospital within 30 days after admission. Analysis of primary outcome will be analyzed using the Wilcoxon rank‐sum test. Results will be presented as medians with interquartile ranges and 95% confidence intervals for the median difference. Inclusion began in June 2023, and the study aims to include 1000 patients from five sites over an estimated time period of 3–4 years. The trial is registered at clinicaltrials.gov (identifier: NCT05895838) and euclinicaltrials.eu (2024‐515997‐28‐00).

**Perspectives:**

Optimizing postresuscitation care through large‐scale randomized trials is essential for improving outcomes of OHCA survivors. The findings from this part of the DANOHCA trial will provide evidence regarding the effects of prophylactic olanzapine in comatose OHCA survivors.

**Trial Registration:** EudraCT no 2016‐003265‐26; EU CTIS no 2024‐515997‐28‐00; ClinicalTrials.gov identifier NCT05895838

## 1 Introduction

In Europe, the incidence of Out‐of‐Hospital Cardiac Arrest (OHCA) is approximately 55 per 100.000 inhabitants, and in Denmark more than 5.000 individuals suffer from OHCA yearly, with most cases having a presumed cardiac etiology. Improved prehospital logistics and bystander‐performed basic life support have considerably improved the outcome over the last decades. However, the 30‐day mortality rate remains above 80% [1]. For unconscious patients with OHCA who are resuscitated successfully and admitted to an intensive care unit (ICU), the 30‐day mortality remains at approximately 50% with the main cause of death being irreversible brain injury [2].

Intensive care following resuscitation from OHCA is based on a set of interventions aimed at circulatory stabilization and mitigation of anoxic brain injury [3, 4]. Patients frequently experience postcardiac arrest syndrome and persistence of the condition that precipitated the cardiac arrest [5]. These patients are often admitted to hospital in an unstable condition necessitating both circulatory and respiratory support.

With the nationwide DANOHCA trial we seek to evaluate four new potential interventions for improving outcomes after OHCA. A separate protocol manuscript will be published for each intervention, with the present manuscript describing the intervention of prophylactic olanzapine compared to placebo for reducing delirium incidence and shortening hospitalization (LINK; Dexamethasone, Wake‐up call, Backrest).

### 1.1 Preventing Delirium With an Antipsychotic Drug

Delirium is an acute, fluctuating, and reversible syndrome characterized by impaired cognition and/or consciousness accompanied by troubles maintaining attention and potential visual and auditory hallucinations [6]. It is the most common sign of acute brain dysfunction among critically ill patients [7]. After awakening, hyper‐ and hypoactive delirium is commonly encountered in all critically ill patients treated with mechanical ventilation in an ICU [8]. Delirium is associated with higher mortality, longer ICU and hospital stays, risk of unplanned removal of devices, and poorer compliance with physical therapy [9, 10]. This is even more pronounced in patients resuscitated after cardiac arrest, where the hypoxic brain injury per se induces brain dysfunction [11]. The optimal management of delirium is uncertain. A study comparing treatment with haloperidol, ziprasidone, or placebo failed to prove benefit of the antipsychotic treatment compared with placebo in critically ill patients with manifest delirium [12]. A more recent trial assessing only haloperidol also failed to prove that haloperidol led to a significantly shorter duration of hospitalization compared to placebo [13].

Limited data exists on prophylactic use of antipsychotics to prevent delirium in critically ill patients, and no studies have tested prophylactic use in OHCA survivors specifically. Prophylactic administration of antipsychotics may be indicated in high‐risk postoperative patients, but there is no evidence currently supporting this approach for OHCA survivors [14, 15]. Olanzapine is an atypical antipsychotic agent of the thienobenzodiazepine class that blocks multiple receptors including dopaminergic, serotonergic, adrenergic, histaminic, and muscarinic. Olanzapine has been suggested to alleviate delirium as well as nausea, vomiting, and pain [16]. Early prophylactic treatment with olanzapine could therefore reduce the number of patients developing delirium, thereby improving the rehabilitation leading to shorter duration of hospitalization.

This part of the DANOHCA trial aims to fill this research gap by evaluating the efficacy of prophylactic use of olanzapine in adults who remain comatose after resuscitation from OHCA. We hypothesize that prophylactic use of olanzapine reduces the number of patients developing delirium, thereby leading to a greater number of days alive and out of the hospital within 30 days in an intention‐to‐treat (ITT) analysis.

## 2 Methods

### 2.1 Trial Design

This study is an investigator‐initiated, randomized, controlled, multicenter clinical trial with co‐enrolment into four different interventions: (1) olanzapine or placebo, (2) dexamethasone or placebo, (3) backrest elevation to 35° or 5°, and (4) early (≤ 6 h) or late (28–36 h) wake‐up call. Intervention‐specific rationale and procedures are described in four corresponding protocol manuscripts (LINK; Dexamethasone, Wake‐up call, Backrest) and a statistical analysis plan (SAP) that outlines the methodological approach to analyzing the common primary, secondary, and tertiary outcomes of the DANOHCA trial (LINK; SAP). Patients and members of the public were not involved in the design of the trial due to the emergency nature of OHCA and the inability of patients to participate in the planning process.

After completion of screening procedures, patients are randomized in a 1:1 fashion to receive either the active intervention or placebo/control intervention for each of the four interventions. The randomization sequence was developed using the PROC PLAN procedure in SAS (SAS Institute Inc., Cary, NC, USA). The randomization is revealed by opening a sealed box containing allocation to backrest positioning, time for wake‐up call, and blinded vials/tablets for dexamethasone and olanzapine respectively, as well as a unique trial identifier and a QR code for data entry into the electronic case report form (eCRF). The study is not stratified by trial site as sealed boxes are prepared centrally and delivered to trial sites randomly upon the inclusion rate.

The DANOHCA trial is registered at clinicaltrials.gov (identifier: NCT05895838) and at the EU CTIS under 2024‐515997‐28‐00, accessible at euclincaltrials.eu (previously registered with EudraCT number: 2021‐005876‐21 at clinicaltrialsregister.eu). The trial has been approved by the Danish Medicines Agency (DKMA) and Danish Medical Research Ethics Committees in accordance with the Clinical Trials Regulation No. 536/2014 and registered with CTIS Trial number 2024‐515997‐28‐00; Transition authorized with conditions on 11‐10‐2024; the current protocol v02‐12‐2025 (SM‐2) was authorized on 05‐12‐2025. Prior to the transition to CTR the trial had been approved by the Danish Medicines Agency no. 2021121966 since 07‐02‐2022 and the regional ethics committee of the Capital Region journal number H‐21077461 since 08‐07‐2022. The full study protocol is available on the study website under the “Trial Documents” section [17].

### 2.2 Trial Conduct

The trial is conducted according to all applicable national and international legislation and guidelines including the revised Declaration of Helsinki [18] and the international Good Clinical Practice guidelines [19]. The trial and protocol were developed in accordance with the International Conference on Harmonization (ICH) guidelines [19, 20, 21] and the Standard Protocol Items: Recommendations for Interventional Trials (SPIRIT) statement [22, 23]. A completed SPIRIT 2025 checklist is provided in Supporting Information. The trial adheres to EU and national legislation on medical research in patients in emergency situations who are temporarily incapacitated and unable to provide informed consent [24, 25, 26, 27]. The trial is approved as emergency research with proxy consent, as the interventions should be started without delay. Informed consent is obtained from an appointed legal representative, and subsequently from the next of kin as soon as possible. Informed consent is obtained from the patient at the earliest opportunity after the patient regains capacity. All consenting parties may withdraw their consent for trial participation at any time. Screening is performed as soon as possible after hospital admission by the physician in charge. The screening log includes all patients admitted to ICU after OHCA with inclusion of the patient's ID, demographic data, and reasons for the exclusion of screened patients (lack of inclusion criteria, presence of exclusion criteria, or logistical reasons).

During ICU‐stay, included patients are screened for delirium three times daily by clinical staff using the delirium screening tool Confusion Assessment Method for the Intensive Care Unit (CAM‐ICU) [28]. Delirium is considered to be present if a patient has a positive CAM‐ICU assessment.

### 2.3 Trial Intervention

Upon ICU admission, patients are randomized in a 1:1 ratio to receive either the active intervention or a matching placebo/control intervention for each of the four trial interventions. Thus, the trial is designed as a 2 × 2 × 2 × 2 factorial randomized trial, with the four interventions reported as separate trials. The balance of allocation across the 16 possible treatment combinations is monitored during generation of the randomization sequence and as part of the planned interim analyses.

For this intervention within the DANOHCA trial, patients will receive either a dose of 10 mg olanzapine (Olanzapin, Stada Arzneimittel AG, Germany) or matching placebo administered by feeding tube upon ICU admission. The patients will receive 10 mg olanzapine or matching placebo the following two evenings at 6.00 p.m. for a total of three doses. If patients wake up and are extubated, the study drug is continued for three administrations unless patients are discharged from the ICU before the final administration. Prior to randomization, patients will be excluded if Long QT Syndrome has been confirmed or suspected because of potential QT‐prolonging effect and concerns for arrhythmia. All patients are monitored by mandatory telemetry of heart rhythm for 96 h or until life sustaining therapies are withdrawn. In the event of delirium, patients are treated according to local practice at the enrolling site, most often including dexmedetomidine, haloperidol, or midazolam.

### 2.4 Blinding

The olanzapine versus placebo intervention is blinded to both clinical personnel, site investigators, and statisticians. The study drug of olanzapine will be delivered by The Hospital Pharmacy of the Capital Region. Packaging into sealed boxes is performed by trained healthcare professionals and will be validated by another staff member before the box is sealed. The study boxes with the olanzapine intervention contain an opaque container with three tablets of either olanzapine or placebo. Preparation of study drugs for administration will be performed by a nurse at the admitting site who is not involved in the clinical care of the patient. The drug administration schedule will be identical for both active medication and placebo and registered both in medical records and study eCRF for monitoring of adherence. During the conduct of the trial, treatment allocation will remain concealed and will only be unblinded in the event of a serious adverse event (SAE) or a suspected unexpected serious adverse reaction (SUSAR) at the discretion of the trial sponsor.

### 2.5 Patient Eligibility and Enrollment

The trial is coordinated and led by The Department of Cardiac Intensive Care, Copenhagen University Hospital—Rigshospitalet, Copenhagen, Denmark. Comatose patients resuscitated after OHCA with a suspected cardiac etiology are enrolled consecutively at five university hospitals representing the five Danish health regions: Copenhagen University Hospital—Rigshospitalet, Aalborg University Hospital, Aarhus University Hospital, Odense University Hospital, and Zealand University Hospital—Koege.

All patients with OHCA admitted to one of the five participating ICUs are screened for inclusion. Eligible OHCA patients fulfilling all inclusion criteria and no exclusion criteria (Table 1 Inclusion and exclusion criteria) are included as soon as possible and no later than 3 h after ROSC. Immediately after inclusion in the trial, a sealed box containing trial medication and allocation group for the two physical interventions is opened, and the four interventions are initiated. Co‐enrollment in other trials must be permitted by the steering group following careful evaluation of the risk of interaction with the four per‐protocol interventions.

**TABLE 1:** Inclusion and exclusion criteria.

Inclusion criteria	Exclusion criteria
All the listed criteria (1–4) must be met: Age ≥ 18 years OHCA of presumed cardiac cause Sustained ROSC^a^ Unconsciousness (GCS < 9) (patients not able to obey verbal commands) after sustained ROSC at the time of randomization	Females of childbearing potential if pregnancy is suspected (unless a negative HCG test can rule out pregnancy within the inclusion window) Known bleeding diathesis (medically induced coagulopathy (e.g., warfarin, NOAC, clopidogrel) does not exclude the patient) Suspected or confirmed acute intracranial bleeding Suspected or confirmed acute stroke Unwitnessed asystole Known limitations in therapy and Do Not Resuscitate‐order Known disease making 180 days survival unlikely Known prearrest cerebral performance category (CPC) 3 or 4 functional status > 3 h (180 min) from ROSC to screening Systolic blood pressure < 80 mmHg despite fluid loading/vasopressor and/or inotropic medication^b^ Use of intra‐aortic balloon pump/axial flow device/ECMO^c^ Temperature on admission < 30°C. Known allergy for dexamethasone or olanzapine Ongoing (within 48 h) treatment with olanzapine or dexamethasone Known back or hip condition that precluded the patients from being positioned with backrest from 0° to 45° angle Known or suspected Long QT Syndrome (LQTS) Known active fungal disease. Localized skin lesions do not exclude patients from inclusion Estimated body weight < 45 kg

*Note:* Since postresuscitation care and intervention are regarded as emergency procedures and should not be delayed, there should only be taken easily accessible sources (previous in‐house chart records, history from emergency medical services) to assess Criteria 2, 5, 6, 7, 8, 12, 13, 14, and 15.

^a^ Sustained ROSC: sustained ROSC is when chest compressions or mechanical circulatory support have been not required for 20 consecutive minutes and signs of circulation persist.

^b^ If the systolic blood pressure (SBP) is recovering during the inclusion window (180 min) the patient may be included.

^c^ If the patient is weaned and the device is removed during the inclusion window (180 min), the patient may be included.

### 2.6 Outcomes

#### 2.6.1 Primary Outcome

The primary outcome for the study is days alive and out of the hospital within 30 days in the two treatment arms, as a modified intention to treat (ITT) analysis. The primary outcome is determined as follows: A day (≥ 24 h) spent at health care facility is counted as time in hospital. Outpatient visits and brief hospitalizations for less than 24 h are not counted as days in hospital. Days admitted to hospital or as deceased within 30 days from randomization are subtracted from 30 days and the result is rounded down to the nearest whole number of days. For patients dying during initial 30 days, the time spent alive outside of hospital is retained for the outcome calculation.

#### 2.6.2 Secondary Outcomes

Secondary outcomes are:

- All‐cause mortality at 90 days.
- Number of CAM–ICU negative days at 30 days.
- Number of days without pharmacological treatment for delirium (other than study drug during the intervention period).
- Number of patients with one or more serious adverse reactions to olanzapine in the ICU.
- Cerebral performance category (CPC) and modified Rankin scale (mRS) at follow‐up 3–6 months after cardiac arrest (conducted either through physical attendance or telephone interview according to participant preference).

#### 2.6.3 Exploratory Outcomes

Exploratory outcomes are as follows:

- Serum neuron‐specific enolase [29] (NSE) at 48 and 72 h [30].
- Number of CAM‐ICU positive patients 48 h after randomization.
- ICU length of stay.
- Hospital length of stay (including in‐patient rehabilitation and transfer to referral hospital).
- Number of patients receiving rescue medication.
- Number of days with rescue medication per patient.

### 2.7 Statistical Methods

#### 2.7.1 General Principles

The four interventions in the DANOHCA trial are considered as four individual trials, and data will be analyzed and reported individually; hence, no adjustment for multiple comparisons is planned for the primary outcome. A full statistical analysis plan is published together with the four separate protocol articles (LINK: SAP). Since previous trials in patients after OHCA predominantly yielded neutral results, all four interventions are accepted without consideration of potential interactions. If more than one intervention demonstrates a nonneutral effect, a post hoc evaluation of possible interactions and potential safety aspects will be conducted and reported. Also, as an investigation of potential safety issues related to possible interactions between the four interventions, we will report interactions in relation to mortality and the occurrence of other SAE. All regression analyses will be adjusted for site as a covariate.

All analyses will be conducted according to the modified ITT principle, including all randomized participants who meet the inclusion criteria and have available outcome data, or imputed outcome data (see Section 2.7.5). Throughout, categorical variables will be presented as counts with proportions, whereas continuous variables will be presented as mean ± SD if normally distributed, and as median (25–75th percentile) if nonnormally distributed, as evaluated by quantile–quantile plots. The primary analysis of the primary outcome will include the modified ITT population. Further, we plan a sensitivity analysis in the per protocol population to assess the effect. The per protocol cohort will be defined as only those trial participants who have received all scheduled doses of the pharmacologic intervention with olanzapine.

#### 2.7.2 Sample Size Considerations

We estimate that 35% of patients will die during the initial hospital course (0 days alive out of hospital) [31]. For patients surviving hospitalization, the expected mean number of days out of hospital will be 15 days; the mean number of days outside hospital will therefore be 9.8 days for the population, with an estimated standard deviation of 7 days. To detect an increase of 1.5 hospital free days with power > 90% and a two‐sided significance level of 5%, a total of 920 patients will be required.

#### 2.7.3 Analysis of Primary Outcome

Days alive outside the hospital within 30 days will be analyzed using the Wilcoxon rank‐sum test between the two treatment arms. Results will be presented as medians with interquartile ranges and 95% confidence intervals for the median difference (calculated using nonparametric bootstrapping with 1000 resamples). Relative differences between days alive outside hospital groups will be reported as percentages, with confidence intervals obtained by bootstrapping. As a prespecified supplementary analysis, days alive outside the hospital within 30 days will also be expressed as the mean between‐group difference with a 95% confidence interval. Sensitivity analyses using mixed‐effects linear regression models with site as a random effect, adjusting for other interventions, will be performed to assess the robustness of the findings.

#### 2.7.4 Analysis of Survival Data (Secondary Outcome)

We will use log‐binomial regression to estimate the relative risk of 90‐day mortality, defined as the proportion of patients who die from any cause between randomization and Day 90. If log‐binomial models are infeasible due to nonconvergence, logistic regression models will be used instead, and odds ratios will be reported instead of relative risk. As additional analyses, Kaplan–Meier curves for each allocation group will be estimated, graphically displayed, and compared by the log‐rank test. Furthermore, Cox proportional hazard models will be applied to estimate hazard ratios with 95% confidence intervals for time to death between treatment groups. As prespecified subgroup analyses, the interaction between treatment allocation and each of the following baseline variables will be assessed: sex, age, time to ROSC, lactate level upon hospital admission, shockable primary rhythm, witnessed cardiac arrest, bystander‐performed cardiopulmonary resuscitation, and presence of STEMI consistent with acute myocardial infarction. Proportional hazards assumptions will be assessed using Schoenfeld residuals and log–log plots. If deviations from the proportional hazards assumption are detected, the relevant covariates will be included with time‐varying HR instead, or if this is not sufficient to solve the deviation, Cox regressions with stratified baseline hazards will be utilized. Further, causes of death (cardiovascular, neurological, multiorgan failure) between the treatment arms will be compared as competing risks with the Nelson–Aalen cumulative incidence method as hypothesis generating. Any changes from the prespecified analysis plan will be reported.

#### 2.7.5 Missing Data

Missing data for the primary outcome is expected to be minimal (< 5%) given the registry‐based follow‐up for mortality. The primary analysis will include all participants with available data. For secondary and tertiary outcomes, missing data mechanisms will be evaluated, and multiple imputation will be used if appropriate.

### 2.8 Substudies

In the trial protocol, several predefined substudies at participating sites are described [17]. From November 2025, neuroinflammatory biomarkers will be measured in jugular venous bulb blood collected via a retrograde central venous catheter (rCVK) in patients enrolled at Copenhagen University Hospital—Rigshospitalet and Odense University Hospital. Paired sampling of arterial blood and jugular bulb blood from the rCVK will be collected to isolate the cerebrovascular bed and calculate the transcerebral release of neurovascular injury and inflammation biomarkers at predefined timepoints 0–12, 24, and 48 h after randomization.

### 2.9 Biobank

A biobank containing blood samples drawn upon arrival in the ICU and daily for up to 72 h after admission has been established. NSE is the only predefined blood‐based biomarker of outcome (secondary outcome) in the main trial, whereas substudies will investigate other markers of brain injury and inflammation. The trial biobank will enable further investigation of novel markers of potential prognostic importance and provide insights into disease mechanisms.

### 2.10 Publication Plan

The findings from each of the four DANOHCA interventions will be reported as separate manuscripts submitted to peer‐reviewed international journals, and all results—whether beneficial, neutral, or harmful—will be published. In accordance with the EU Clinical Trials Regulation, summary results will also be publicly accessible through the EU Clinical Trials Information System (CTIS) and automatically posted on the EU Clinical Trials Register within 1 year of the end of the trial. The statistical analysis plan will be published before the last patient is included in the trial.

### 2.11 Safety and Adverse Events

#### 2.11.1 Adverse Events (AE)

Comatose OHCA patients requiring postresuscitation intensive care have a high risk of morbidity and mortality and thus, a high probability of AE. Therefore, the following events will not be considered an AE in the present study, as they are frequently occurring in OHCA patients during ICU stay: myocardial infarction (unless a de novo), minor bleeding, metabolic disorder, and other biochemical abnormalities.

AEs are categorized according to the following definitions: Major bleeding, infections (pneumonia, sepsis, pyelonephritis, upper airway infections), renal impairment requiring renal replacement therapy, electrolyte disorders, metabolic disorders (hypoglycemia, prolonged hyperglycemia), cardiac events (ventricular fibrillation, ventricular tachycardia, atrial fibrillation requiring cardioversion, new need for pacing, prolonged QT interval > 560 ms, circulatory collapse), respiratory events (self‐extubation, need for reintubation), seizures (tonic–clonic, myoclonic, status epilepticus), extrapyramidal side effects, skin complications (pressure ulcers, acne, atrophy, purpura, reduced wound healing), and death after withdrawal of life‐sustaining therapy. The following AE are considered potentially related to the olanzapine intervention (adverse reaction, AR): ventricular arrhythmias, prolonged QT interval, bucco‐linguo‐masticatory syndrome. For each AE report, there will be an additional question on whether it constitutes a SAE/serious adverse reaction (SAR), defined as an AE/AR that results in death, is life‐threatening, requires prolongation of hospitalization, or results in significant disability. SUSARs will be reported by the sponsor to the EudraVigilance database as soon as possible and no later than 7 days after registration.

#### 2.11.2 Monitoring of AE and SAE

Patients are monitored for the development of an AE for 30 days after randomization or until hospital discharge if this occurs later than 30 days. A prespecified form in the eCRF allows for daily recording of AE during ICU‐stay. AE occurring after discharge from the ICU and within 30 days from randomization will be evaluated at the time of ambulatory follow‐up (scheduled for 3–6 months after cardiac arrest). AE will only be reported to the trial sponsor if they can be classified as a SAE with a suspected direct relation to the trial intervention. Once a year all SAEs occurring at the trial centers will be submitted and a safety report of all trial patients will be submitted to the competent authorities as required. All SAEs will be reported as outcome measures in the final trial report. Monitoring of the trial is performed by the Danish Good Clinical Practice (GCP) units according to preplanned monitoring plans at the participating sites.

#### 2.11.3 Interim Analyses

An Independent Data Monitoring Committee (IDMC) consisting of three members with expertise in cardiac arrest research and intensive care was appointed before the start of the trial. The committee is responsible for safeguarding the interests of trial participants, assessing the safety and efficacy of the interventions during the trial, and monitoring the overall conduct of the clinical trial following a charter agreed upon before trial commencement. An interim analysis with de‐identified data for safety based on the respective primary outcomes for the four interventions and the occurrence of the protocol specified categories of SAE was performed after inclusion of 500 patients out of the preplanned 1000 patients. The interim analysis was performed as a safety review without formal efficacy or futility testing by the IDMC, resulting in no remarks.

### 2.12 Trial Status and Timeline

Randomization began in June 2023, and patient inclusion is currently proceeding according to the expected timeline, with the last patients of a total of 1000 patients expected to be included in July 2026. Follow‐up is expected to be completed in October 2026.

## 3 Discussion

The DANOHCA trial will evaluate four commonly used and potentially beneficial interventions in comatose OHCA patients admitted to the ICU. The evidence for each intervention is sparse, and the purpose of the trial is to evaluate whether these four interventions will reduce morbidity and mortality associated with PCAS, including the impact of prophylactic olanzapine. Preventing delirium in OHCA patients is essential because it is associated with prolonged ICU stay, higher mortality, and long‐term cognitive impairment. Despite the high prevalence of delirium among this group of patients, there is a lack of effective preventive treatments. Prevention of delirium may improve neurological recovery and quality of life as well as reduce healthcare costs.

The present intervention will provide insights into the effect of prophylactic treatment with olanzapine in preventing delirium, potentially leading to shorter hospitalization. The results could inform ICU guidelines for delirium management in comatose cardiac arrest patients and potentially improve the standard of care.

### 3.1 Strengths

The trial has several strengths. It is a nationwide, investigator‐initiated, multicenter trial testing commonly used treatments, all with conflicting or limited evidence. The trial has a pragmatic design making it feasible and easy to embed in routine clinical practice. Automated randomization and blinding of the olanzapine versus placebo intervention to both the clinical personnel, site investigators, and statisticians minimizes the risk of bias. The risk of side effects and complications is likely to be limited, as olanzapine is a well‐known drug given within normal dosage regimens for a short period of time. The use of a validated diagnostic tool for the assessment of delirium may increase the generalizability of the results. In the event of delirium, the trial design allows for treatment according to local practices at the enrolling site, ensuring appropriate care for patients who develop delirium.

Finally, the assumptions regarding outcomes after cardiac arrest are likely to be realistic, as the sample size is based on previous data from our department [31]. With 920 participants, the trial provides more than 90% power to detect a 1.5‐day difference in days alive and out of the hospital at 30 days. This is a clinically meaningful and patient‐centered outcome.

### 3.2 Limitations

The trial also has limitations. Restricting enrollment to a single country with a public healthcare system may limit global external validity, though representative standards of care can mitigate this. During ICU stay, screening for delirium will be performed routinely, while different local guidelines regarding screening of delirium on the wards at the enrolling sites may result in patients not being screened for delirium on a regular basis after ICU discharge. Although a false‐positive screening with the CAM‐ICU is rare, it may result in unnecessary pharmacological or nonpharmacological treatment.

The trial is not powered to evaluate interactions between the four interventions. The study design allows independent randomization and analysis for each intervention, preserving validity of the primary hypothesis within each domain, under the clinical assumption of no interaction. Although exploratory cross‐arm interaction analyses are planned—mainly to assess potential synergistic or antagonistic effects—they are acknowledged as hypothesis‐generating only.

## 4 Conclusion

The DANOHCA trial aims to evaluate new interventions to improve outcomes for OHCA patients. We will evaluate if prophylactic olanzapine has implications for the number of days alive and out of the hospital within 30 days of comatose OHCA patients undergoing intensive care after resuscitation. Due to its design as an investigator‐initiated, randomized, multicenter trial with a large sample size, the trial is expected to provide conclusive results that may inform clinical practice.

## Author Contributions

J.K., J.E.M., H.S., J.B.A., B.S.R., A.M.G., S.C., L.P.K.A., S.M., M.A.S.M., and C.H. designed the study protocol of the DANOHCA trial. Together with S.M. these authors wrote the statistical analysis plan. B.L. and S.M. wrote the first draft of the manuscript. All authors contributed to manuscript editing and approved the final version of the manuscript.

## Funding

The trial has received unrestricted research grants from the Novo Nordisk Foundation (NNF22OC0079649), the Danish Heart Foundation (2023‐12401), and the Central Denmark Region Health Research Foundation. The funding agencies had no role in the design or conduct of the study.

## Conflicts of Interest

J. Kjaergaard reports grant from the Novo Nordisk Foundation (NNF22OC0079649, present trial). Further, J. Kjaergaard reports grant from Sundhedsdonationer (2025‐0271); and speaker's honorarium from Vingmed A/S and Sundhedsdonationer. C. Hassager reports grants from the Lundbeck Foundation (R186‐2015‐2132), the Novo Nordisk Foundation (NNF20OC0064043), and The Danish Heart Foundation; and speaker's honorarium from Abiomed and BD. J.E. Moeller reports Institutional grants from the Novo Nordisk Foundation and Abiomed; speakers fee from Abbott and Abiomed; and advisory board from Boston Scientific and Magenta. All outside the submitted work. The other authors declare no conflicts of interest.

## Supporting information


**Data S1:** aas70289‐sup‐0001‐Supinfo.docx.

## Data Availability

The data that support the findings of this study are available from the corresponding author upon reasonable request.
